# Oscillation of mineral compositions in Core SG-1b, western Qaidam Basin, NE Tibetan Plateau

**DOI:** 10.1038/srep32848

**Published:** 2016-09-14

**Authors:** Xiaomin Fang, Minghui Li, Zhengrong Wang, Jiuyi Wang, Jiao Li, Xiaoming Liu, Jinbo Zan

**Affiliations:** 1 Institute of Tibetan Plateau Research, Chinese Academy of Sciences (CAS), Beijing 100085, China; 2 Key Laboratory of Continental Collision and Plateau uplift, Institute of Tibetan Plateau Research, CAS, Beijing 100085, China; 3 CAS Center for Excellence in Tibetan Plateau Earth Sciences, Beijing, 100101, China; 4 Key Laboratory of Tibetan Environment Changes and Land Surface Processes, CAS, Beijing 100085, China; 5 Department of Earth and Atmospheric Sciences, City College of New York, CUNY, New York, NY 10031, USA; 6 University of Chinese Academy of Sciences, Beijing 100049, China

## Abstract

Uplift of the Tibetan Plateau since the Late Miocene has greatly affected the nature of sediments deposited in the Qaidam Basin. However, due to the scarcity of continuously dated sediment records, we know little about how minerals responded to this uplift. In order to understand this response, we here present results from the high-resolution mineral profile from a borehole (7.3–1.6 Ma) in the Basin, which shows systematic oscillations of various evaporite and clay minerals that can be linked to the variation of regional climate and tectonic history. In particular, x-ray diffraction (XRD) analyses show that carbonate minerals consist mainly of calcite and aragonite, with minor ankerite and dolomite. Evaporates consist of gypsum, celesite and halite. Clay minerals are principally Fe-Mg illite, mixed layers of illite/smectite and chlorite, with minor kaolinite and smectite. Following implications can be drawn from the oscillations of these minerals phases: (a) the paleolake was brackish with high salinity after 7.3 Ma, while an abrupt change in the chemical composition of paleolake water (*e.g.* Mg/Ca ratio, SO_4_^2−^ concentration, salinity) occurred at 3.3 Ma; (b) the three changes at ~6.0 Ma, 4.5–4.1 Ma and 3.3 Ma were in response to rapid erosions/uplift of the basin; (c) pore water or fluid was Fe/Mg-rich in 7.3–6.0 Ma, Mg-rich in 6.0–4.5 Ma, and K-rich in 4.1–1.6 Ma; and (d) evaporation rates were high, but weaker than today’s.

Minerals, and especially evaporative and clay minerals, provide important geological archives for studying sedimentary responses to environmental changes^1^,^2^,^3^,^4^,^5^. They have been widely used to reconstruct paleoclimate, paleoenvironment, sedimentary weathering, provenance and tectonic events. Evaporative minerals, which are commonly found in desert basins, indicate the hydrogeochemical conditions present at the time of their precipitation. Different evaporative mineral assemblages can reflect the chemical weathering of different rock lithologies, the mixing of inflow waters from several sources, and various hydrochemical and physical processes. Clay minerals are important constituents of lacustrine and marine sediments. Their occurrence is dictated by clay reaction mechanisms, evolution of porewater chemistry and crystal-chemical stability fields^4^,^6^,^7^,^8^, and therefore, they can also be a proxy for changing climates. For example, the degree of the smectite to illite reaction has frequently been used as a geothermometer to reconstruct the thermal and tectonic history of sedimentary basins^6^,^9^. Much of the available mineral data are from marine cores, whereas there is a shortage of long-term mineral data from arid inland Asian regions, and especially from the Qaidam Basin (QB), the largest intermontane basin of the Northeast Tibetan Plateau (NE TP).

The Qaidam Basin (Fig. 1) is the result of intracontinental deformation and plateau uplift due to the collision of the Indian and Eurasian plates^10^,^11^, and is currently filled with up to 12,000 m of thick, continuous Cenozoic sediments^12^. The western QB has experienced significantly strong deformation, accompanied by the uplift of the surrounding mountains to the current average elevations of over 4000–5000 meters above sea level (MASL)^12^,^13^,^14^. During the Paleocene-Eocene, most sedimentation occurred in the western part of the Basin^15^, whereas after the Oligocene, the Basin was completely enclosed, forming a huge lake around the depocenter. After the Pliocene, the depocenter was separated by faults, and developed into sub-basins with a variety of different sedimentary characteristics, due to the acceleration in the rates of mountain uplift and compression^15^. This unique sedimentary sequence and its location in the transitional zone between the arid Asian interior and the East Asian Monsoon regions not only make it an ideal place to study the high-resolution records of long-term climate and erosion histories^16^, but also provide important information on tectonic deformation processes and the potential linkage of tectonic uplift to climate change.

As part of a joint Sino-German project, a 723m-deep core, named SG-1b, was recovered from the top of the Jianshan Anticline (38°21′9.46″N, 92°16′24.72″E, Fig. 1) with an average recovery rate of ~93%. Paleomagnetic dating of the core yielded an age range of ~7.3–1.6 Ma, with a sediment accumulation rate (SAR) of 6.5 to 30.4 cm/ka^16^. According to Lu^17^, the lower section of the core (723–245 m, ca. 7.3–3.6 Ma) is characterized by gray to dark-gray fine siltstones with striking horizontal millimeter-scale laminae; the middle section (245–200 m, ca. 3.6–3.3 Ma) is characterized by massive bedding and occasionally weak laminations accompanied by coarse siltstones and thin bands of sandstones; and the upper section (200–0 m, ca. 3.3–1.6 Ma) contains abundant millimeter- to centimeter-scale interbedded sandstones and scattered gypsum crystals. Ooid carbonates begin to occur at 81 m (2.6 Ma) and occur more frequently upwards.

Here we present a 6-Ma (*i.e*. 7.3–1.6 Ma) long high-resolution profile of the mineral compositions (including evaporates, carbonates and clay minerals) recorded in Core SG-1b. We show that the Core’s minerals have indeed documented important climate events in the region’s paleoenvironmental history.

## Results

### Carbonate minerals

XRD analyses show that carbonate minerals in the core are composed of calcite, aragonite, dolomite and ankerite. The abundance of calcite ranges from 1% to 70%, while that of aragonite spans from 2 to 84%. Both calcite and aragonite abundances decrease dramatically at a depth of 206 m (3.3 Ma), suggesting changes in the chemical composition of the paleolake water. The occurrences of dolomite ((Ca, Mg)(CO_3_)_2_) and ankerite (Ca(Fe, Mg, Mn)(CO_3_)_2_) in the core are discontinuous (Fig. 2). The abundance of dolomite in most samples is <10%, except at 111 m (2.7 Ma), 271–284 m (3.8–3.9 Ma), 351–359 m (4.5–4.6 Ma), and 419 m (5.0 Ma), where dolomite contents are higher than ~30% of all carbonates. The CaCO_3_ and MgCO_3_ molar abundance in dolomite are ~45–61% and ~39–55%, respectively, based on XRD analysis, where d (104) = 2.88–2.9285^18^ (Fig. 2), reflecting the changing chemical composition of dolomite. Ankerite appears mainly at depths of 72–206 m (2.4–3.3 Ma), 288–307 m (3.9–4.2 Ma), 367–437 m (4.4–5.2 Ma), and 516–723 m (6.2–7.3 Ma), and abundance is mostly <10%.

Calcite is the major carbonate phase present throughout the profile. The crystallinity of calcite was recorded using the full-width half-maximum (FWHM) function, and XRD of 3.03 Å (104) gave values ranging from 0.165 to 0.306. Small amounts of Mg are often integrated into the calcite lattice. The MgCO_3_ content of calcite in the profile ranges from 0 to 3.32% (Fig. 2), suggesting that these calcites are Mg-poor.

### Sulfate minerals and halite

Sulfate minerals are discontinuous in the core. Most gypsum (CaSO_4_·2H_2_O) appears at depths of 0–33.4 m (1.6–1.9 Ma), 135–279 m (2.9–3.1 Ma), 507–521 m (6.2–6.3 Ma) and 582–721 m (6.6–7.3 Ma), with the majority exhibiting an abundance of no more than 7%. High gypsum contents occur at 3.3 Ma, 3.1–2.9 Ma and 1.9 Ma. Trace amounts of anhydrite occur at a depth of 571 m (6.6 Ma) and upwards to the top of the core, indicating its occurrence is possibly a result of transformation from gypsum.

Celestite (SrSO_4_) appears at depths of 7–163 m (1.7–3.1 Ma), 243–297 m (3.6–4 Ma), 377–527 m (4.7–6.3 Ma), 582–616 m (6.6–6.8 Ma), and 658–720 m (7.1–7.3 Ma). It is not an abundant component of the sediments found in the core, usually exhibiting contents of <10%. However, high contents of celestite occur at 4.1–3.6 Ma, 2.8 Ma, 2.5 Ma, 2.0 Ma and 1.8 Ma.

Most halite appears from 585 m (6.6 Ma) upward, with content ranging between 1–10%, gradually increasing upward (Fig. 2). Halite crystallinity (HC), represented by the integral breadth of the 2.82 Å peak, ranges from 0.22 to 0.39, with a mean value of 0.27 (Δ°2θ, Fig. 2).

### Silicate/clay minerals

Clay mineral assemblages consist of Fe-Mg illite (25–63%), mixed layers of illite/smectite (13–64%), chlorite (4–20%; Mg-chlorite only between 6.0–4.1 Ma), kaolinite (<8%), and smectite (usually <11%, except at 550 m (~6.4 Ma), where it is ~30%, and at 705 m (~7.2 Ma), where it is ~25% (Fig. 3). The chlorite and illite abundances exhibit similar patterns in their variations, but these patterns are in turn different from those of kaolinite, smectite and mixed-layer illite/smectite (I/S; Fig. 3). All illites are rich in Fe and Mg (Fig. 3). The illite crystallinity (IC), represented by the integral breadth of the 10 Å peak, ranges from 0.24 to 0.44, with a mean value of 0.37 (Δ°2θ, Fig. 3). Almost all IC in the core occurs in the anchizone (280–360 °C)^19^,^20^, but some limited IC is found in the diagenetic zone (<280 °C)^19^,^21^ (Fig. 3), indicating that burial diagenesis mainly occurred between 6.0–4.5 Ma, and that low-grade thermal metamorphism took place in the Core. The IC values gradually increases between 7.3–6.0 Ma and 2.9–1.6 Ma, but are highly variable between 6.0–4.1 Ma, and stable between 4.1–2.9 Ma.

Similarly, chlorite crystallinity (CC), as represented by the integral breadth of the 7 Å peak, ranges from 0.28 to 0.56, with a mean of 0.45 (Δ°2θ, Fig. 3). The CC values are high and stable between 7.3–6.0 Ma, variable between 6.0–4.1 Ma, and low and stable between 4.1–1.6 Ma. Fe-Mg chlorite occurs within the 7.3–6.2 Ma and 4.1–1.6 Ma periods, and Mg-chlorite between 6.2–4.1 Ma (Fig. 3). A statistically significant correlation exists between IC and CC when R^2^ = 0.58, suggesting temperature exerts an influence on CC (since IC is controlled by temperature).

XRD analyses suggest mixed-layer illite/smectite minerals (a thermodynamically metastable intermediate product between smectite and illite)^22^ in the Core exhibit disorder (R_0_ I/S) and order (R_3_ I/S) (Fig. 3). Because there is no distinct boundary in the XRD spectrum between smectite and I/S, or between I/S and illite, most R_3_ I/S identified by XRD could also be R_1_ I/S^9^. Moreover, the R_1_ I/S is more stable than any other mixed layer I/S phase because it has a unique structure, composition, and ordering pattern^9^,^23^,^24^. Thus, any clay mineral identified as R_0_ I/S by XRD is more likely to be a mixture of discrete smectite and R_1_ I/S, *i.e.* mixed-layer I/S with R > 1 is a mixture of R1 I/S and illite^9^.

## Discussion

### Origins

#### Carbonate minerals

The precipitation of carbonate minerals in typical modern lakes follows a low-Mg calcite → high-Mg calcite → aragonite sequence, accompanied by an increased Mg/Ca ratio in lake water. Most aragonite and some calcite should have been directly deposited by the paleolake water, *e.g.* when the salinity of a solution reached ~1.8 times the concentration of modern seawater^2^. However, in the Core, some calcite and trace amounts of dolomite are not authigenic, because suspended carbonates were found to contain calcite (5–21%) and trace amounts of dolomite (SI Table), presumably originating from the physical weathering of carbonate rocks and older carbonate sediments in the source region.

Dolomite can form as a primary precipitate, a diagenetic replacement, or out of hydrothermal fluid. With high permeability, a high fluid flow rate is possible. This facilitates the supply of Mg, essential to the formation of dolomite^3^. Bacterial metabolism may aid dolomite precipitation in settings where sulfate-reducing bacteria flourish^3^. The chemical composition of dolomite can be further modified during burial and metamorphism, in response to changing environmental parameters, including temperature, pressure, burial history, chemistry of pore fluid, and co-precipitating mineral phases. Because of its complex origins and formation conditions, it is difficult to identify climatic information based on dolomite alone. The formation of ankerite (Ca(Fe, Mg, Mn)(CO_3_)_2_) has been related to the Fe-Mg rich interstitial water/pore water present during the transformation of clay minerals. The varying abundance of ankerite can therefore reflect the variable availabilities of Fe, Mg and Ca in fluids (interstitial water/pore water).

#### Sulfates and halite

**Figure 1:**
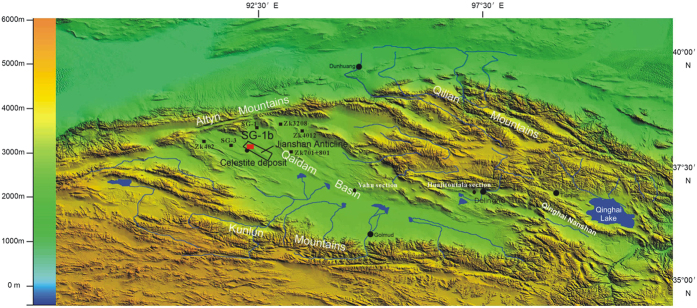
Map showing the Qaidam Basin, the location of the study area, as well as other area for comparison. It was generated using ArcGIS 9.2 based on DEM data from NASA (http://reverb.echo.nasa.gov/reverb/).

When the salinity of a solution reaches approximately five times the concentration of modern seawater, gypsum precipitates from brine^2^. The Sr in the celestite could have originated from three possible sources: 1) the weathering of rocks; 2) Sr-rich hydrothermal fluids; and 3) pore fluids. We may assume this because a celestite deposit which has been proposed to be of hydrothermal origin^25^ occurs near the Core, in the QB (Fig. 1). During the process of calcite dissolution-reprecipitation, there is a net input of strontium into pore fluids that are oversaturated by celestite^26^. As one of the most common evaporative minerals, halite crystals should directly precipitate from brine and pore water when its salinity reaches 11 or 12 times that of modern seawater^2^.

#### Clay minerals

Generally, clay minerals in lake sediments are lithogenous as by-products of the physical and chemical weathering of rocks, *e.g*. chlorite and illite form under severe physical weathering conditions, and kaolinite and smectite form when chemical weathering conditions predominate. However, significant quantities of kaolinite and smectite appear in the modern sediments of Lake Nella and soils in Antarctica, a very cold region^27^,^28^, which would suggest that the presence of kaolinite and smectite is not always indicative of a warm climate and/or chemical weathering conditions. A weak correlation between abundance of kaolinite and smectite (R^2^ = 0.23, Fig. 4a) also suggests their origins to be different. Despite that the sediments deposited in the Jianshan Anticline area were mainly transported there by fluvial process and wind action from the Altyn Mountains to the northwest^17^, most of the clay minerals in the Core would have then undergone thermal metamorphism, with a few that have experienced diagenesis (Fig. 3). Therefore, there could be other origins, such as an authigenic precipitation, and a transformation between the clay minerals. The formation of kaolinite possibly depends on the contents of Mg and Ca, while the formation of smectite relies on the Si, Ca, Mg and Fe contents in the lake/pore water^28^. The significant correlation between illite and chlorite (R^2^ = 0.62, Fig. 4b), and between illite and I/S (R^2^ = −0.88, Fig. 4c), would suggest that most illite was transformed from I/S, and some from physical weathering with chlorite. Similarly, chlorite can also be transformed from kaolinite as well as from weathering processes. In the Core, there are likely to be a couple of explanations for this: (1) there was a significant relation between chlorite and kaolinite (R^2^ = 0.56) (Fig. 4d); and (2) kaolinite content decreases as burial depth increases. The formation of authigenic chlorite is related to the presence of Fe-Mg rich interstitial water/pore water, while K-rich water favors the formation of illite and I/S mixed minerals^29^,^30^. The ions required for the formation of these minerals were perhaps derived from the breakdown of detrital Fe-Mg minerals and lacustrine sediments during deposition or early burial^5^,^31^. Despite that original clay mineral assemblages resulting from weathering have also been preserved in the Core^31^, it was difficult to quantify the proportion of original clays.

On the other hand, temperature is an important control of the crystallinity and chemistry of illite and chlorite, the formation of I/S mixed minerals, and the switch from disorder I/S to order I/S^5^,^6^,^7^,^8^,^9^,^32^,^33^,^34^. For example, low IC is preserved in the cold climate sections. IC in the anchizone of the Core indicates that the low-grade thermal metamorphic temperature ranged from 280–360 °C^19^,^21^ (Fig. 3). The S-I reaction is an abiotic dissolution re-crystallization process that typically requires temperature conditions of 300–350 °C^9^. The transformation temperature from disorder R_0_ I/S to order R_1_ I/S is ~100–110 °C^32^,^33^, and R_1_ I/S is stable between ~150 °C and ~225 °C^34^. The Fe-Mg contents of illite and chlorite will increase with increasing temperature^6^. Mg-chlorite occurs at temperatures of >100 °C^5^,^7^.

### Implications for environmental changes

The oscillation of mineral compositions in the Core implies significant environmental changes during 7.3–1.6 Ma in the western Qaidam Basin, especially at ~6.0 Ma, ~4.5–4.1 Ma and ~3.3 Ma. Climate change and/or tectonic activity could explain these mineral oscillations. A dry climate could have allowed the direct precipitation of minerals in water with high ionic concentrations. Intense faulting caused by tectonic activity could have facilitated a great inflow of hydrothermal fluid containing Fe-Mg ions at high heat, affecting especially clay minerals and ankerite. This certainly occurred in the QB, where the presence of hydrothermal fluids was widespread^1^,^15^,^23^.

The presence of gypsum and celestite indicates that the paleolake water was brackish, with high concentrations of SO_4_^2−^ after 7.3 Ma. The gradual increase in halite content after 6.6 Ma suggests an increasing salinity in the paleolake water, probably caused by a dry regional climate and high rates of evaporation (Fig. 3). Possible dry-cold events happened at 6.6 Ma and 2.9 Ma as the lower IC and variability of evaporates (Fig. 3). Grain sizes and lithofacies in the Core SG-1b and fossils in QB also suggest that the climate was dry but much wetter than the Quaternary during 7.3–3.6 Ma^17^. Climatic proxies suggest that rapid drying began principally from 8 Ma in the eastern QB (Huaitoutala section, Fig. 1)^35^, and the climate had already been dry after 5.3 Ma in the central QB (Yahu Section, Fig. 1)^36^ with a drought event at 3.6 Ma in the western and central QB (Core SG-1b and Yahu Section, Fig. 1)^17^,^36^ and a long-term stepwise drying trend in the western QB since 3.1 Ma (Core SG-1, SG-3 Fig. 1)^37^,^38^. The widespread distribution of evaporative minerals in the western QB also suggested drought climate since late Pliocene (SI Figure)^16^,^38^,^39^. Arid fluctuating climate controlled the depositional environment in the north-central QB after 3.1 Ma^40^. The marked drying of northwest China (including QB) was possible triggered by (a) the high elevation of the TP (probably ~1500 m at ~ 8–5 Ma)^41^ and the surrounding mountains of the QB^42^,^43^ and (b) Miocene global cooling and Pliocene–Pleistocene rapid global cooling^35^,^44^. The high elevation has made it difficult for moist air to enter the QB. Global cooling decreased evaporation rates and water vapor concentrations in the atmosphere.

**Figure 2:**
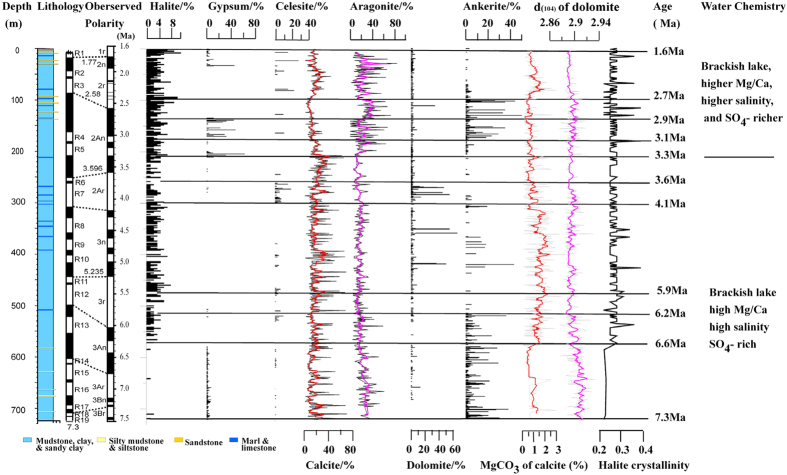
Variations in halite, gypsum, celestite, calcite, aragonite, dolomite and ankerite contents. The magnetostratigraphy of the SG-1b core was derived from Zhang *et al*.^15^,^16^. Colored lines represent a five-point running average.

**Figure 3:**
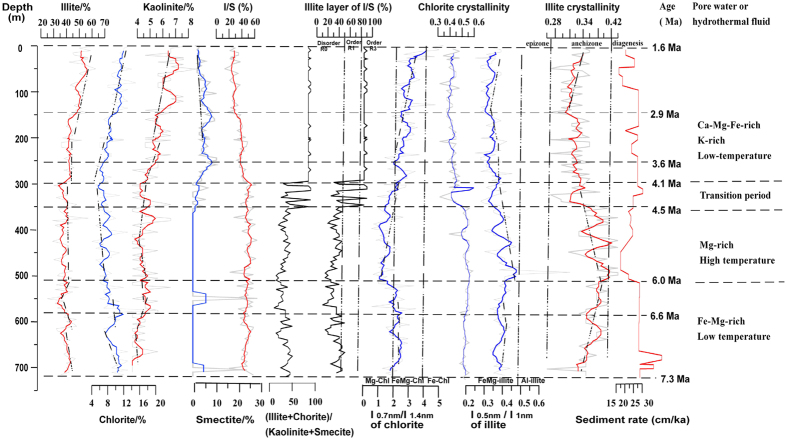
Variations in: the clay minerals of illite, chlorite, kaolinite, smectite, illite/smectite (I/S); illite (% of I/S); chlorite crystallinity (Δ°2θ); the chemical composition of chlorite (I_0.7nm_/I_1.4nm_); illite crystallinity (Δ°2θ); and the chemical composition of illite (I_0.5nm_/I_1.0nm_). The sedimentation rate was derived from Zhang *et al*.^16^. Colored lines represent a five-point running average.

**Figure 4:**
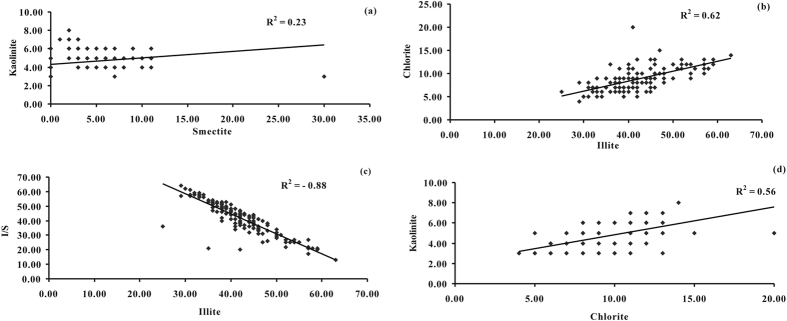
Correlations between (**a**) the abundance (%) of smectite and kaolinite; (**b**) illite and chlorite; (**c**) illite and I/S; (**d**) chlorite and kaolinite.

Three major changes of mineral properties have been observed in the core at ~6.0 Ma, ~4.5–4.1 Ma and ~3.3 Ma. At ~6.0 Ma, the significant change in the relative abundance of ankerite, the values of IC and CC, the type of chlorite and the Fe-Mg content of illite (Figs 2 and 3), all indicate an abrupt shift in the nature of thermal fluid and/or pore-water—changing from the relatively low temperatures in 7.3–6.0 Ma to the relatively high temperatures between ~6.0–4.5 Ma, and changing from the Fe-Mg-rich fluid/pore water in 7.3–6.0 Ma to the Mg-rich fluid/pore water between ~6.0–4.5 Ma. During the 4.5–4.1 Ma transition period, corresponding to the most important stage of uplift of the NE TP at that time^45^, the category of I/S mixed minerals turns from disorder to order (Fig. 3), and the value of (illite + chlorite)/(kaolinite + smectite) decreases dramatically. These changes indicate that the chemistry of the fluid or pore water was different between 4.1–1.6 Ma from between 7.3–4.5 Ma (Fig. 3). At ~3.3 Ma, the significant change in the relative abundance among calcite, aragonite, and gypsum, and a weak increase in halite abundance signify an abrupt change in the chemical composition of the paleolake water (*e.g.* the Mg/Ca ratio, SO_4_^2−^ concentration, salinity). This agrees with the variations in granulometric parameters and lithofacies features, which suggest that the paleolake was shallow at ~3.3 Ma as a result of the rapid and continuous uplift of the Jianshan Anticline^17^.

The three observed changes at ~6.0 Ma, ~4.5–4.1 Ma and ~3.3 Ma are likely to be related with the rapid erosion and high SAR values noted in Core SG-1b at >7.3–6.0 Ma, 5.2–4.2 Ma and 3.6–2.6 Ma (Fig. 3). These high SAR values can be linked directly to the tectonic evolution of the Qaidam Basin^16^. The tectonic deformation and uplift event at 8~6 Ma has been reported from the QB and from various parts of the TP^45^,^46^,^47^. The main and intense uplift of the northwestern QB happened at 5.3–4.5 Ma^45^,^47^, and rapid uplift happened at ~3.6 Ma^12^. Tectonic activity not only triggered a drought climate, but also resulted in the transportation of materials into the Basin^35^,^48^, a great inflow of hydrothermal fluid, and therefore high SAR rates. The similar variation of SAR among the cores in the western QB (SI Figure) also provided an evidence for the tectonic activities. Therefore, climatic change and uplift history of QB controlled the depositional environment and oscillations of minerals in the Core SG-1b.

## Materials and Methods

Mineral compositions in the Core were examined at 1 m intervals using an X-ray diffractometer (XRD) (a Rigaku D/MAX-2000: Cu, Ka1, 1.5406 Ǻ, 40 kV, 40 mA). A subsection of these samples were treated with ultrapure water at 2 m intervals, then diluted with HCl and H_2_O_2_ to remove dissolvable ions, calcium carbonate and organic matter; the clay mineral fraction (<2 *μ*m) was then separated in a centrifuge. For each sample, this fraction was then scanned using XRD, to include the sequential collection of three XRD patterns, *i.e.* under natural (air-dried) conditions (N), after saturation with ethylene-glycol for 24 hrs in desiccators (EG), and after being heated in a muffle furnace at 490 °C for 2 hrs (H). Diffraction patterns (2θ) were scanned from 3° to 30°, with a step size of 0.07°. Minerals were identified by peak area. The measurements were conducted at the Micro Structure Analytical Laboratory (MSAL), Peking University, in compliance with Chinese oil and gas industry standard SY/T 5163-2010.

The MgCO_3_ content in the calcite was deduced from the deviation of the XRD (104) using the following calibration^18^:


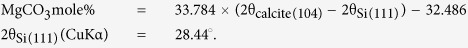


## Additional Information

**How to cite this article**: Fang, X. *et al*. Oscillation of mineral compositions in Core SG-1b, western Qaidam Basin, NE Tibetan Plateau. *Sci. Rep.* **6**, 32848; doi: 10.1038/srep32848 (2016).

## Supplementary Material

Supplementary Information
