# Supplementary material for: Oscillation of mineral compositions in Core SG-1b, western Qaidam Basin, NE Tibetan Plateau
Source: Sci Rep. 2016 Sep 14;6:32848. doi: 10.1038/srep32848 (PMC5021985; doi:10.1038/srep32848)
Supplement: Supplementary Information [file srep32848-s1.doc]

# Oscillation of mineral compositions in Core SG-1b, western Qaidam Basin, NE Tibetan Plateau

**Xiaomin Fang**^a,b,c^**, Minghui LI** ^a,d*^**, Zhengrong Wang**^e^**, Jiuyi Wang**^a,b^**, Jiao LI** ^a,b, f^**, Xiaoming Liu**^a,b^**, Jinbo Zan**^a,b^

^a^ Institute of Tibetan Plateau Research, Chinese Academy of Sciences (CAS), Beijing 100085, China

^b^ Key Laboratory of Continental Collision and Plateau uplift, Institute of Tibetan Plateau Research, CAS, Beijing 100085, China

^c^ CAS Center for Excellence in Tibetan Plateau Earth Sciences, Beijing, 100101

^d^ Key Laboratory of Tibetan Environment Changes and Land Surface Processes, CAS, Beijing 100085, China

^e^ Department of Earth and Atmospheric Sciences, City College of New York, CUNY, New York, NY 10031

^f^ University of Chinese Academy of Sciences, Beijing 100049, China

*corresponding author Minghui Li: Email: [liminghui@itpcas.ac.cn](mailto:liminghui@itpcas.ac.cn)

Table Suspended minerals of river water in Qaidam Basin

| Sample No. | Location | Quartz | Mica / illite | Chlorite | Plagloclase | Microcline | Calcite | Amphibole | Dolomite |
| --- | --- | --- | --- | --- | --- | --- | --- | --- | --- |
| 1 | N35°41'2.64"  E 94°2'55.38" | 39% | 16% | 16% | 17% | 5% | 6% | - |  |
| 2 | N 39°26'11.34"  E 94°59'53.94" | 22% | 13% | 19% | 17% | 7% | 11% | 11% |  |
| 3 | N 35°53'18.48"  E 94°23'32.70" | 41% | 11% | 16% | 18% | 3% | 11% | - |  |
| 4 | N 35°52'27.72"  E 94°34'5.52" | 34% | 20% | 46% | - | - | - | - |  |
| 5 | N 39°26'23.58"  E 95° 3'54.96" | 29% | 20% | 31% | - | - | 20% | - |  |
| 6 | N 39°21'15.48"  E 95°16'5.28" | 16% | 14% | 24% | 26% | 20% | - | - |  |
| 7 | N 39°22'51.54"  E 94°19'13.14" | 25% | 10% | 12% | 19% | 8% | 20% | 6% |  |
| 8 | N 39°26'0.78"  E 95°10'30.54" | 19% | 15% | 38% | 10% | 10% | 8% | - |  |
| 9 | N37°22'14.64"  E 98°50'32.88" | 16% | 20% | 33% | 31% | - | - | - |  |
| 10 | N38°27'57.48"  E99°32'8.82" | 20% | 14% | 30% | 23% | - | 12% | - |  |
| 11 | N 38°14'31.80"  E 99° 9'36.66" | 20% | 21% | 31% | 27% | - | - | - |  |
| 12 | N 38°25'4.32"  E99°31'48.72" | 26% | 14% | 19% | 11% | 6% | 6% | 17% |  |
| 13 | N38°42'35.40"  E 99°29'44.40" | 22% | 16% | 38% | 8% | - | - | - |  |
| 14 | N 38°46'47.70"  E 99°31'31.98" | 26% | 11% | 21% | 10% | 3% | 5% | - | 5% |
| 15 | N 38°21'14.16"  E 99°19'47.88" | 15% | 15% | 22% | 25% | 23% | - | - |  |
| 16 | N 38°48'26.46"  E100°10'52.62" | 15% | 19% | 27% | 11% | 7% | 21% | - |  |
| 17 | N 38°13'3.54"  E102°45'13.02" | 28% | 12% | 11% | 12% | 5% | 7% | 11% | 14% |
| 18 | N 39°32'52.08"  E95°41'22.26" | 16% | 5% | 12% | 19% | 16% | 11% | 9% | 12% |
| 19 | N35°52'  E94°34' | 38% | 7% | 11% | 13% | 6% | 9% | 8% |  |
| 20 | N38°13'  E102°45' | 28% | 16% | 21% | 10% | 6% | 19% | - |  |


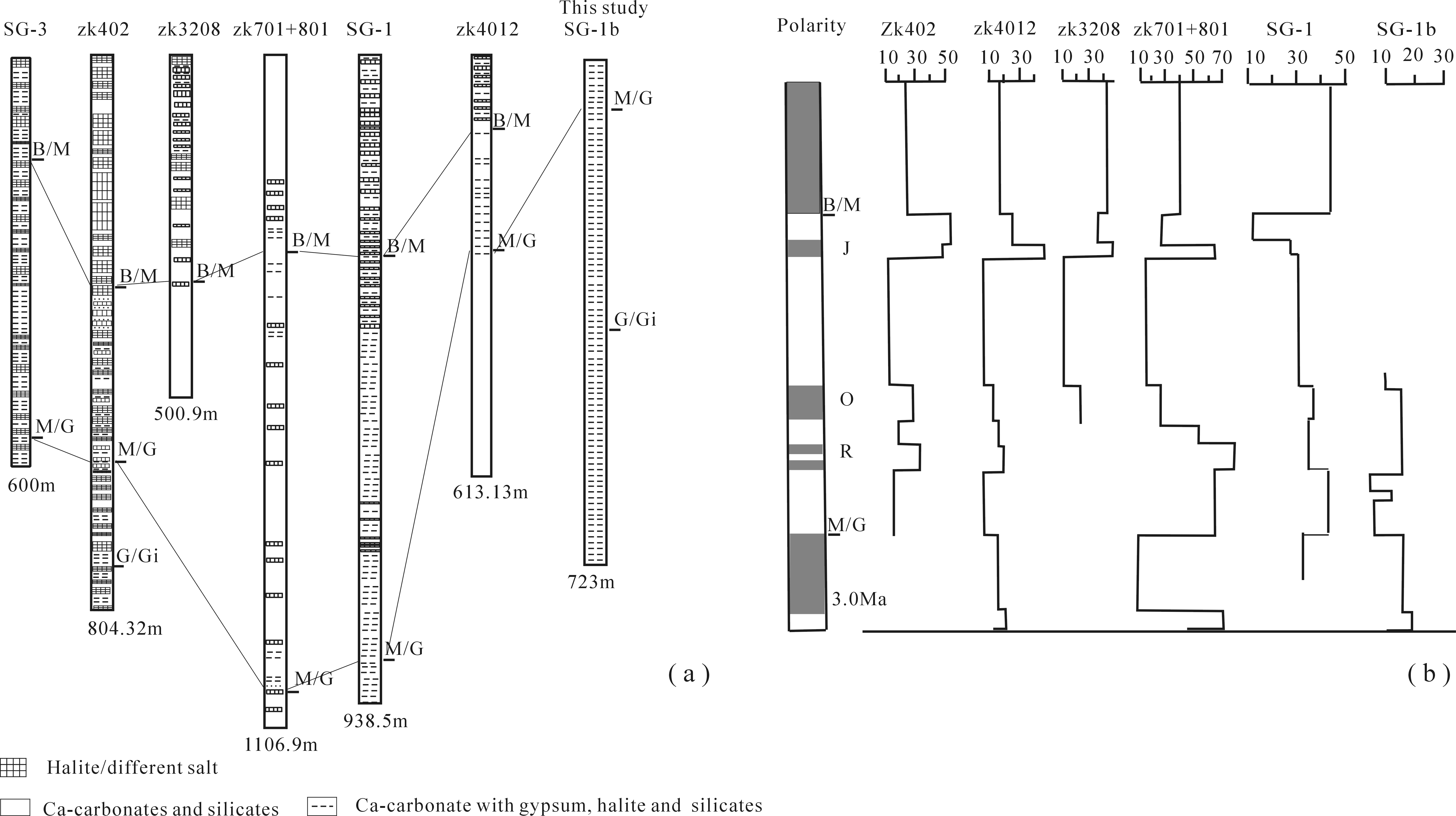


**SI Figure**

(a)Distributions of evaporates of cores in the western QB. (b)Sediment accumulation rate (SAR, cm/ka) and Paleomagnetic polarity of cores in the western QB. B: Brunhes; M: Matuyama; G: Gauss epochs; Gi: Gilbert. They are after Zhang et al. (2014)^16^, Cai et al.(2012)^38^ and Shen et al. (1993)^39^.
